# Terpenoids from the Octocoral *Sinularia gaweli*

**DOI:** 10.3390/ijms160819508

**Published:** 2015-08-18

**Authors:** Wun-Jie Lin, Tung-Ying Wu, Tzu-Rong Su, Zhi-Hong Wen, Jih-Jung Chen, Lee-Shing Fang, Yang-Chang Wu, Ping-Jyun Sung

**Affiliations:** 1 Graduate Institute of Marine Biology, National Dong Hwa University, Pingtung 944, Taiwan; E-Mail: abcdewq213@hotmail.com; 2 National Museum of Marine Biology & Aquarium, Pingtung 944, Taiwan; 3 Chinese Medicine Research and Development Center, China Medical University Hospital, Taichung 404, Taiwan; E-Mail: kuma0401@gmail.com; 4 Department of Beauty Science, Meiho University, Pingtung 912, Taiwan; E-Mail: a081002@mail.tsmh.org.tw; 5 Antai Medical Care Cooperation Antai Tian-Sheng Memorial Hospital, Pingtung 928, Taiwan; 6 Department of Marine Biotechnology and Resources, Asia-Pacific Ocean Research Center, National Sun Yat-sen University, Kaohsiung 804, Taiwan; E-Mail: wzh@mail.nsysu.edu.tw; 7 Graduate Institute of Pharmaceutical Technology & Department of Pharmacy, Tajen University, Pingtung 907, Taiwan; E-Mail: jjchen@mail.tajen.edu.tw; 8 Department of Sport, Health and Leisure, Cheng Shiu University, Kaohsiung 833, Taiwan; E-Mail: lsfang@csu.edu.tw; 9 School of Pharmacy, College of Pharmacy, China Medical University, Taichung 404, Taiwan; 10 Center for Molecular Medicine, China Medical University Hospital, Taichung 404, Taiwan; 11 Graduate Institute of Natural Products, Kaohsiung Medical University, Kaohsiung 807, Taiwan

**Keywords:** *Sinularia gaweli*, eudesmane, cembrane, octocoral, iNOS

## Abstract

Two eudesmane sesquiterpenoids, verticillatol (**1**) and 5α-acetoxy-4(14)-eudesmene-1β-ol (**2**) and two cembrane diterpenoids, (–)-leptodiol acetate (**3**) and sinulacembranolide A (**4**) were isolated from the octocoral *Sinularia gaweli* and compounds **2**–**4** are new isolates. The structures of new terpenoids **2**–**4** were elucidated by spectroscopic methods and by comparison the spectral data with those of known analogues. Terpenoid **4** was found to inhibit the accumulation of the pro-inflammatory inducible nitric oxide synthase (iNOS) protein of the lipopolysaccharide (LPS)-stimulated RAW264.7 marcophage cells.

## 1. Introduction

**Figure 1:**
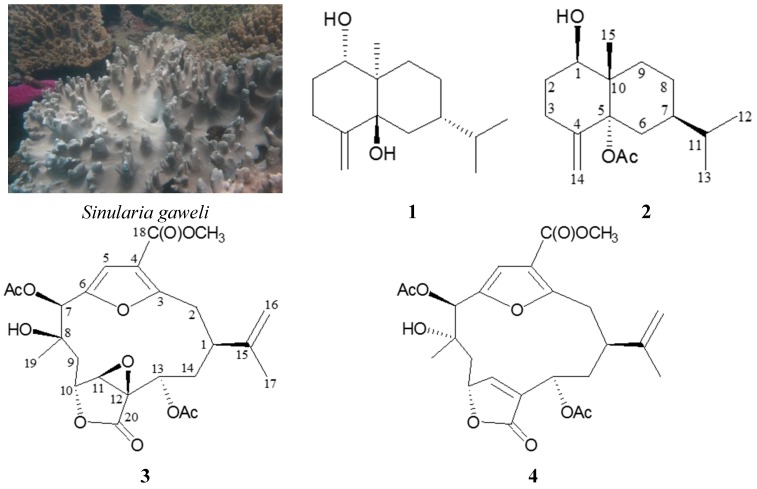
The soft coral *Sinularia gaweli* and the structures of verticillatol (**1**); 5α-acetoxy-4(14)-eudesmene-1β-ol (**2**); (–)-leptodiol acetate (**3**); and sinulacembranolide A (**4**).

Octocorals belonging to the *Sinularia* genus have been well-recognized as marine organisms containing large quantities of terpenoid metabolites that exhibit varying degrees of bioactivities [1,2]. Our previous investigation on the soft coral *Sinularia gaweli* (Verseveldt, 1978, phylum Cnidaria, class Anthozoa, order Alcyonacea, family Alcyoniidae). (Figure 1) had afforded norcembranoidal diterpenes and steroid analogues [3,4,5]. Our continuing studies on this soft coral has, again, led to the isolation of four terpenoid metabolites, including two eudesmane sesquiterpenoids, verticillatol (**1**) [6] and 5α-acetoxy-4(14)-eudesmene-1β-ol (**2**) and two cembranes, (–)-leptodiol acetate (**3**) and sinulacembranolide A (**4**) (Figure 1), and compounds **2**–**4** are new isolates. In this paper, the isolation, structure determination and anti-inflammatory activity of compounds **1**–**4** are described.

## 2. Results and Discussion

A known eudesmane sesquiterpenoid, verticillatol (**1**), was obtained in this study. This compound had been obtained from a Vietnam plant *Litsea verticullata* [6]. The NMR data and rotation value of **1** were identical to those of verticillatol described previously.

5α-Acetoxy-4(14)-eudesmene-1β-ol (**2**) was isolated as a colorless oil and the molecular formula for this compound was determined to be C_17_H_28_O_3_ (four units of unsaturation) using HRESIMS (C_17_H_28_O_3_ + Na, *m*/*z* 303.19293, calculated 303.19307). Comparison of the ^13^C NMR and distortionless enhancement by polarization transfer (DEPT) data with the molecular formula indicated that there was an exchangeable proton, which required the presence of a hydroxy group. This deduction was supported by a broad absorption in the IR spectrum at 3465 cm^–^^1^. The IR spectrum also showed a strong band at 1735 cm^–^^1^, consistent with the presence of an ester group. From the ^1^H and ^13^C NMR spectra (Table 1), **2** was found to possess an acetoxy group (δ_H_ 2.03, 3H, s; δ_C_ 168.9, C; 21.8, CH_3_). An additional unsaturated functionality was indicated by ^13^C resonances at δ_C_ 111.6 (CH_2_-14) and 145.1 (C-4), suggesting the presence of an exocyclic carbon-carbon double bond. Thus, the proposed skeleton of **2** was suggested to be a bicyclosesquiterpenoid.

**Table 1:** ^1^H (400 MHz, CDCl_3_) and ^13^C (100 MHz, CDCl_3_) NMR data, ^1^H–^1^H COSY and HMBC correlations for sesquiterpenoid **2**.

Position	δ_H_ Multiplicity (*J* in Hz)	δ_C_, Multiplicity	^1^H–^1^H COSY	HMBC
1	4.00 dd (12.0, 4.8)	72.9, CH	H_2_-2	C-2, -10, -15
2α	1.85 m	30.9, CH_2_	H-1, H-2β, H_2_-3	n. o.
β	1.55 m	–	H-1, H-2α, H_2_-3	C-1, -3
3α	2.22 m	30.2, CH_2_	H_2_-2, H-3β	C-1, -2, -4, -5, -14
β	1.26 m	–	H_2_-2, H-3α	n. o.
4	–	145.1, C	–	–
5	–	87.3, C	–	–
6α	2.61 br d (14.4)	28.5, CH_2_	H-6β	C-5, -10
β	1.41 dd (14.4, 12.4)		H-6α, H-7	C-7, -8
7	1.26 m	38.6, CH	H_2_-6, H_2_-8, H-11	C-9
8α	1.22 m	23.6, CH_2_	H-7, H-8β, H_2_-9	C-7, -9
β	1.59 m	–	H-7, H-8α, H_2_-9	n. o.
9	1.70 m	30.4, CH_2_	H_2_-8	C-7, -8, -15
10	–	43.3, C	–	–
11	1.47 m	32.5, CH	H-7, H_3_-12, H_3_-13	C-8
12	0.90 d (6.8)	19.6, CH_3_	H-11	C-7, -11, -13
13	0.89 d (6.8)	19.9, CH_3_	H-11	C-7, -11, -12
14a	5.05 s	111.6, CH_2_	H-14b	C-3, -5
b	4.90 s	–	H-14a	C-3, -4, -5
15	0.79 s	12.3, CH_3_	–	C-1, -5, -9, -10
5-OAc (5^a^) (the C=O)	–	168.9, C	–	–
5-OAc (5^b^) (the Me)	2.03 s	21.8, CH_3_	–	C-5^a^

n. o. = not observed; COSY = correlation spectroscopy; HMBC = heteronuclear multiple bond connectivity; 5^a^ = acetate carbonyl of 5-acetoxy group; 5^b^ = acetate methyl of 5-acetoxy group.

**Figure 2:**
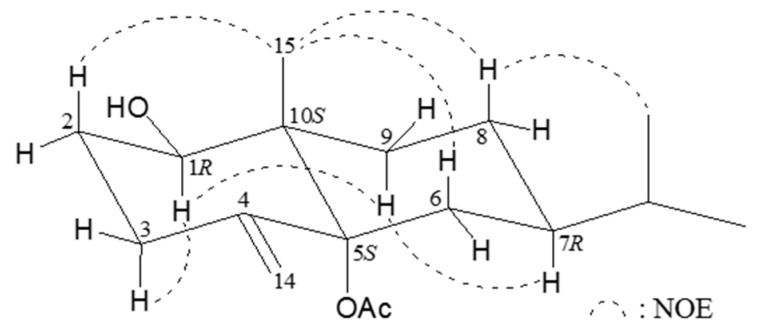
Important nuclear overhauser effect spectroscopy (NOESY) correlations for compound **2**.

From the ^1^H-^1^H COSY and HMBC spectrum of **2** (Table 1), permitted elucidation of the main eudesmane carbon skeleton. The relative configuration of **2** was elucidated by means of a NOESY (nuclear overhauser effect spectroscopy) experiment (Figure 2). The NOEs of H-9α to H-1 and H-7α revealed the hydroxy group at C-1 and isopropyl group at C-7 to be β-oriented. The NOEs between H_3_-15/H-2β, H_3_-15/H-6β, and H_3_-15/H-8β assigned the methyl group at C-10 as β. By comparison of the rotation value of compound **2** (${\text{[α]}}_{\text{D}}^{25}$ +75 (*c* 0.93, MeOH); ${\text{[α]}}_{\text{D}}^{25}$ +25 (*c* 0.93, CHCl_3_)) with those of **1** (verticillatol) (${\text{[α]}}_{\text{D}}^{25}$ −41.2 (*c* 0.13, CHCl_3_)) [6] and the enantiomer of **1**, 4(15)-eudesmene-1β,5α-diol (${\text{[α]}}_{\text{D}}^{23}$ +108 (*c* 0.8, MeOH)) [7] (In this reference, the alphabetical orders for C-14 and C-15 in this compound should be exchanged from the IUPAC recommendation), suggested that the acetoxy group at C-5 should be α-oriented. Since the absolute configuration of 4(15)-eudesmene-1β, 5α-diol had been determined by modified Mosher’s method [7], we were able to assign the absolute configurations of all the chiral centers of **2** as 1*R*, 5*S*, 7*R*, 10*S*. Based on the above findings, the structure of **2** was, therefore, determined to be 5α-acetoxy-4(14)-eudesmene-1β-ol.

The spectral (^1^H, ^13^C NMR and IR) data of **3** were in full agreement with those of a known cembrane analogue, leptodiol acetate, which was isolated from a Panama gorgonian coral identified as *Leptogorgia* sp. [8]. However, the optical rotation value of **3** (${\text{[α]}}_{\text{D}}^{25}$ −24 (*c* 0.33, CHCl_3_); ${\text{[α]}}_{\text{D}}^{25}$ −10 (*c* 0.33, CH_2_Cl_2_)) was substantially different from that of leptodiol acetate (${\text{[α]}}_{\text{D}}^{20}$ +27 (*c* 0.49, CH_2_Cl_2_)); indicating that cembranoid **3** is an enantiomer of leptodiol acetate and assigned as (−)-leptodiol acetate.

**Figure 3:**
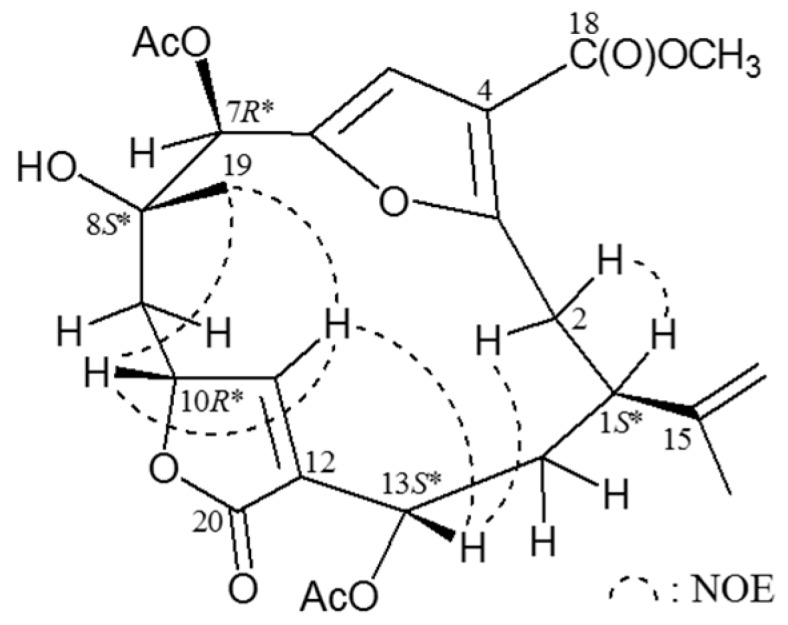
NOESY (nuclear overhauser effect spectroscopy) correlations of compound **4**.

The molecular formula for cembranoid **4** (sinulacembranolide A) was determined to be C_25_H_30_O_10_ (11 units of unsaturation) using HRESIMS (C_25_H_30_O_10_ + Na, *m*/*z* 513.17338, calcd. 513.17312). Absorption for hydroxy and carbonyl groups at 3481, 1756, and 1721 cm^–1^ were observed in the IR spectrum. The ^13^C NMR and DEPT spectra of **4** (Table 2) showed the presence of 25 carbon signals assigned to 5 × CH_3_ (one methoxy group, two from acetyl groups), 4 × CH_2_ (one olefinic), 6 × CH (two olefinics and three oxymethines) and 10 quaternary carbons (four carbonyls and five olefinics). ^1^H and ^13^C NMR data of **4** (Table 2) were similar with those of **3**, particularly the chemical shifts for 11,12-epoxy group in **3** (δ_H_ 4.10, 1H, br s, H-11; δ_C_ 63.0, CH-11; 59.0, C-12) were replaced by a carbon-carbon double bond (δ_H_ 6.16, 1H, d, *J* = 1.2 Hz, H-11; δ_C_ 154.0, CH-11; 129.8, C-12) for molecule **4**. Connectivity information obtained from 2D NMR, including ^1^H–^1^H COSY and HMBC experiments, unambiguously determined the planar structure of **4** (Table 2). The relative configuration of **4** was elucidated from NOESY correlations (Figure 3). In the NOESY experiment for **4**, it was found that one of the methylene protons at C-2 (δ_H_ 2.82) exhibited a correlation with H-1, but not with H-13 and, therefore, it was assigned as H-2α, and the other C-2 proton (δ_H_ 3.57) as H-2β. H-13 showed correlations with H-2β and H-11, but not with H-1, and H-11 showed a correlation with H-10, as well as a small coupling (*J* = 1.2 Hz) detected between H-10 and H-11, indicating the dihedral angle between H-10 and H-11 is approximately 90° and the geometry of the C-11/12 carbon-carbon double bond was *Z* form and C-10 possessing an relative configuration R*-form. H_3_-19 correlated with H-10 and H-11, but not with H-7, indicating that Me-19 and H-7 were β- and α-oriented at C-8 and C-7, respectively. From the above evidence, the relative configuration of chiral carbons of **4** were assumed to be 1S*, 7R*, 8S*, 10R*, and 13S*.

To the best of our knowledge, the C-7, C-8 vicinal diol-based cembranoid analogues are rarely found [9]. In previous studies on *S. gaweli* had afforded an interesting norcembranoid, sinulanorcembranolide A [3], and the biosynthetic pathway for sinulanorcembranolide A was proposed in a later study [10]. This observation is in agreement with the observation that cembrane diterpenoids from Alcyonacea have opposite configuration at C-1 compared to those obtained from Gorgonacea [11].

**Table 2:** ^1^H (400 MHz, CDCl_3_) and ^13^C (100 MHz, CDCl_3_) NMR data, ^1^H–^1^H COSY and HMBC correlations for cembrane **4**.

Position	δ_H_ Multiplicity (*J* in Hz)	δ_C_, Multiplicity	^1^H–^1^H COSY	HMBC
1	2.32 ddd (10.8, 10.8, 3.2)	41.5, CH	H_2_-2, H_2_-14	C-17
2α	2.82 d (15.2)	31.9, CH_2_	H-2β	C-1, -3, -4, -14, -15
β	3.57 dd (15.2, 10.8)	–	H-1, H-2α	C-1, -14
3	–	160.9, C	–	–
4		115.5, C	–	–
5	6.65 d (0.8)	109.1, CH	H-7	C-3, -4, -6
6	–	149.2, C	–	–
7	5.54 br s	76.1, CH	H-5	C-5, -6, -8, -9, -19, -7^a^
8	–	72.6, C	–	–
9α	1.91 dd (14.8, 11.2)	42.9, CH_2_	H-9β, H-10	C-7, -8, -10
β	2.67 dd (14.8, 5.6)	–	H-9α, H-10	C-7, -8, -10, -11
10	4.95 ddd (11.2, 5.6, 1.2)	77.6, CH	H_2_-9, H-11	C-9, -11, -12
11	6.16 d (1.2)	154.0, CH	H-10	C-10, -12, -13, -20
12	–	129.8, C	–	–
13	5.52 dd (11.2, 4.8)	66.7, CH	H_2_-14	C-11, -12, -14, -13^a^
14α	2.63 ddd (11.2, 11.2, 3.2)	36.1, CH_2_	H-1, H-13, H-14β	n. o.
β	1.90 ddd (11.2, 10.8, 4.8)	–	H-1, H-13, H-14α	C-1, -2, -12, -13
15	–	148.4, C	–	–
16	4.82 br s	110.6, CH_2_	H_3_-17	C-1, -15, -17
17	1.81 s	20.7, CH_3_	H_2_-16	C-1, -15, -16
18	–	163.5, C	–	–
19	1.47 s	20.1, CH_3_	–	C-7, -8, -9
20	–	169.5, C	–	–
7-OAc (7^a^) (the C = O)	–	169.6, C	–	–
7-OAc (7^b^) (the Me)	2.17 s	20.9, CH_3_	–	C-7^a^
13-OAc (13^a^) (the C = O)	–	170.3, C	–	–
13-OAc (13^b^) (the Me)	1.99 s	21.0, CH_3_	–	C-13^a^
18-OCH_3_	3.88 s	51.9, CH_3_	–	C-18

n. o. = not observed; 7^a^ = acetate carbonyl of 7-acetoxy group; 7^b^ = acetate methyl of 7-acetoxy group; 13^a^ = acetate carbonyl of 13-acetoxy group; 13^b^ = acetate methyl of 13-acetoxy group.

**Figure 4:**
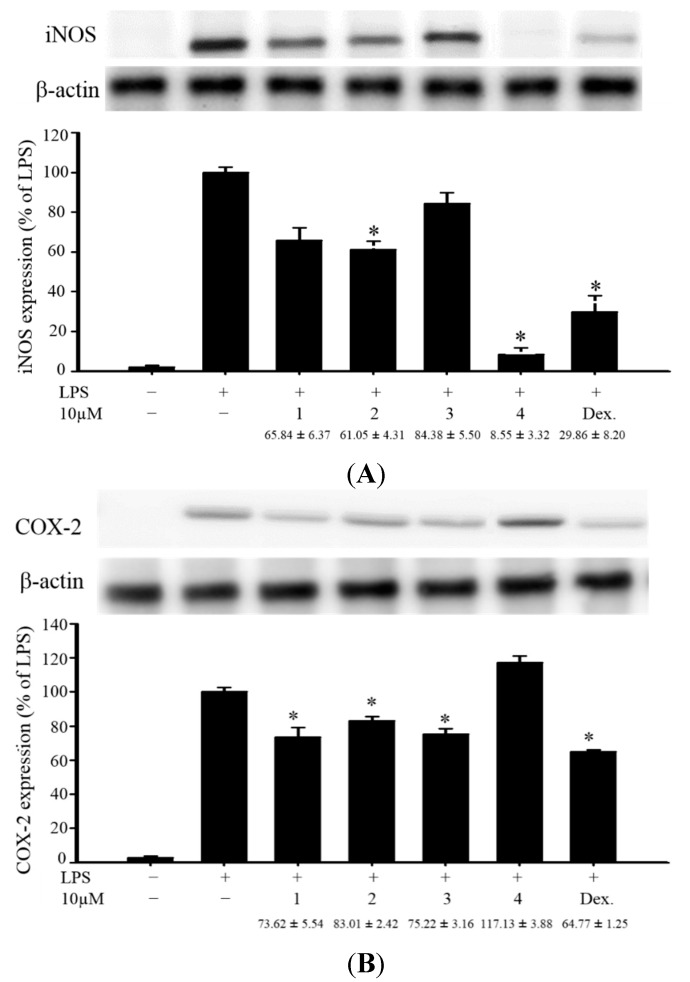
Effects of compounds **1**–**4** on pro-inflammatory iNOS and COX-2 protein expression in LPS-stimulated murine macrophage cell line RAW264.7. (**A**) Relative density of iNOS immunoblot; (**B**) relative density of COX-2 immunoblot. The relative intensity of the LPS-stimulated group was taken to be 100%. Band intensities were quantified by densitometry and are indicated as the percent change relative to that of the LPS-stimulated group. Compound **4** and dexamethasone (Dex.) significantly inhibited LPS-induced iNOS protein expression in macrophage. The experiment was repeated three times. (* *p* < 0.05, significantly different from the LPS-stimulated group).

In the *in vitro* anti-inflammatory activity test, the upregulation of the pro-inflammatory inducible nitric oxide synthase (iNOS) and cyclooxygenase-2 (COX-2) proteins expression of LPS (lipopolysaccharide) -stimulated RAW264.7 macrophage cells was evaluated using immunoblot analysis. At a concentration of 10 μM, compound **4** was found to significantly reduce the levels of iNOS to 8.55% ± 3.32%, relative to the control cells stimulated with LPS only (Figure 4). Thus, compound **4** might be promising as an anti-inflammatory agent, as this compound did not exhibit cytotoxicity to RAW264.7 macrophage cells.

## 3. Experimental Section

### 3.1. General Experimental Procedures

Optical rotation values were measured with a Jasco P-1010 digital polarimeter (Japan Spectroscopic Corporation, Tokyo, Japan). IR spectra were recorded on a Jasco FT-4100 FT-IR spectrometer (Japan Spectroscopic Corporation, Tokyo, Japan); peaks are reported in cm^−1^. NMR spectra were recorded on a Varian Mercury Plus 400 NMR spectrometer (Varian Inc., Palo Alto, CA, USA) using the residual CHCl_3_ signal (δ_H_ 7.26 ppm) as the internal standard for ^1^H NMR and CDCl_3_ (δ_C_ 77.1 ppm) for ^13^C NMR. Coupling constants (*J*) are given in Hz. ESIMS and HRESIMS were recorded using a Bruker 7 Tesla solariX FTMS system (Bruker, Bremen, Germany). Column chromatography was performed on silica gel (230–400 mesh, Merck, Darmstadt, Germany). TLC was carried out on precoated Kieselgel 60 F_254_ (0.25 mm, Merck, Darmstadt, Germany); spots were visualized by spraying with 10% H_2_SO_4_ solution, followed by heating. Normal-phase HPLC (NP-HPLC) was performed using a system comprised of a Hitachi L-7110 pump (Hitachi Ltd., Tokyo, Japan) and a Rheodyne 7725 injection port (Rheodyne LLC, Rohnert Park, CA, USA). Two normal-phase columns (Supelco Ascentis^®^ Si Cat #: 581514-U, 25 cm × 10 mm and 581515-U, 25 cm × 21.2 mm, 5 μm, Sigma-Aldrich, St. Louis, MO, USA) were used for HPLC. Reverse phase HPLC (RP-HPLC) was performed using a system comprised of a Hitachi L-2130 pump (Hitachi Ltd., Tokyo, Japan), a Hitachi L-2455 photodiode array detector (Hitachi Ltd., Tokyo, Japan), a Rheodyne 7725 injection port (Rheodyne LLC., Rohnert Park, CA, USA). A reverse phase column (Supelco Ascentis^®^ Si Cat #: 581343-U, 25 cm × 10.0 mm, 5 μm, Sigma-Aldrich, St. Louis, MO, USA) was used for RP-HPLC.

### 3.2. Animal Material

Specimens of the octocoral *Sinularia gaweli* (Verseveldt, 1978) were collected by hand using self-contained underwater breathing apparatus (SCUBA) equipment off the coast of Sansiantai, Taitung county, Taiwan on 7 May 2013, and stored in a freezer (−20 °C) until extraction. A voucher specimen (NMMBA-TWSC-13031) was deposited in the National Museum of Marine Biology & Aquarium, Taiwan.

### 3.3. Extraction and Isolation

Sliced bodies of *Sinularia gaweli* (wet weight 2794 g, dry weight 756 g) were extracted with ethyl acetate (EtOAc). The EtOAc layer (20.5 g) was separated on silica gel and eluted using *n*-hexane/EtOAc (stepwise, 100:1→99:1→95:5→9:1→4:1→7:3→3:2→1:1→2:3→3:7→1:4→1:9→pure EtOAc, each fraction × 2 L) to yield 21 fractions, A–U, by thin layer chromatography (TLC) analysis. Fraction G (collected from the fraction eluted using *n*-hexane/EtOAc 4:1) was chromatographed on NP-HPLC using a mixture of *n*-hexane and acetone (4:1) to afford nine fractions, G1–G9. Fraction G5 was separated by NP-HPLC using a mixture of dichloromethane (DCM) and acetone (50:1, flow rate: 1.0 mL/min) to afford **2** (2.8 mg, *t*_R_ = 38 min). Fraction H (collected from the fraction eluted using *n*-hexane/EtOAc 7:3) was chromatographed on NP-HPLC using a mixture of *n*-hexane and EtOAc (3:1) to afford 14 fractions, H1–H14. Fraction H9 was separated by NP-HPLC using a mixture of *n*-hexane and acetone (5:1, flow rate: 0.5 mL/min) to afford **1** (0.5 mg, *t*_R_ = 80 min). Fraction N (collected from the fraction eluted using *n*-hexane/EtOAc 1:1) was separated on silica gel and eluted using *n*-hexane and acetone (stepwise, 6:1–1:1) to yield 16 fractions, N1–N16. Fraction N11 was separated by on NP-HPLC using a mixture of *n*-hexane and acetone (2:1) to afford **3** (6.6 mg, *t*_R_ = 23 min). Fraction P (collected from the fraction eluted using *n*-hexane/EtOAc 2:3) was chromatographed on NP-HPLC using a mixture of *n*-hexane and acetone (2:1) to afford 12 fractions, P1–P12. Fraction P7 was separated by RP-HPLC using a mixture of methanol and H_2_O (60:40, flow rate: 3.0 mL/min) to afford **4** (1.4 mg, *t*_R_ = 120 min).

Verticillatol (**1**): colorless oil; ${\text{[α]}}_{\text{D}}^{26}$ −113 (*c* 0.17, CHCl_3_) (reference [5], ${\text{[α]}}_{\text{D}}^{25}$ −41.2 (*c* 0.13, CHCl_3_)); IR (neat) ν_max_ 3444 cm^−1^; ^1^H and ^13^C NMR data were found to be in full agreement with those reported previously [6].

5α-Acetoxy-4(14)-eudesmene-1β-ol (**2**): colorless oil; ${\text{[α]}}_{\text{D}}^{25}$ +75 (*c* 0.93, MeOH) (${\text{[α]}}_{\text{D}}^{25}$ +25 (*c* 0.93, CHCl_3_)); IR (neat) ν_max_ 3465, 1735 cm^−1^; ^1^H (400 MHz, CDCl_3_) and ^13^C (100 MHz, CDCl_3_) NMR data, see Table 1; ESIMS: *m*/*z* 303 [M + Na]^+^; HRESIMS: *m*/*z* 303.19293 (calcd for C_17_H_28_O_3_ + Na, 303.19307).

(–)-Leptodiol acetate (**3**): white solid; mp 183–185 °C; ${\text{[α]}}_{\text{D}}^{25}$ −24 (*c* 0.33, CHCl_3_) (${\text{[α]}}_{\text{D}}^{25}$ −10 (*c* 0.33, CH_2_Cl_2_)); IR (neat) ν_max_ 3502, 1785, 1734 cm^−1^; ^1^H and ^13^C NMR data were found to be in full agreement with those reported previously [8]; ESIMS: *m*/*z* 529 [M + Na]^+^; HRESIMS: *m*/*z* 529.16777 (calcd for C_25_H_30_O_11_ + Na, 529.16803).

Sinulacembranolide A (**4**): white solid; mp 120–123 °C; ${\text{[α]}}_{\text{D}}^{25}$ −10 (*c* 0.07, CHCl_3_); IR (neat) ν_max_ 3481, 1756, 1721 cm^−1^; ^1^H (400 MHz, CDCl_3_) and ^13^C (100 MHz, CDCl_3_) NMR data, see Table 2; ESIMS: *m*/*z* 513 [M + Na]^+^; HRESIMS: *m*/*z* 513.17338 (calcd for C_25_H_30_O_10_ + Na, 513.17312).

### 3.4. In Vitro Anti-Inflammatory Assay

Murine macrophage (RAW264.7) cell line was purchased from ATCC. *In vitro* anti-inflammatory activity of compounds **1**–**4** was measured by examining the inhibition of lipopolysaccharid (LPS)-induced up-regulation of pro-inflammatory inducible nitric oxide synthase (iNOS) and cyclooxygenase-2 (COX-2) protein expression in macrophage cells using Western blotting analysis [12,13,14]. Briefly, inflammation in macrophages was induced by incubating them for 16 h in a medium containing only LPS (10 ng/mL) without compounds. For anti-inflammatory activity assay, compounds **1**–**4** and dexamethasone (10 μM) were added the cells 10 min before LPS challenge. The cells then underwent Western blot analysis. The immunoreactivity data are calculated with respect to the average optical density of the corresponding LPS-stimulated group. For statistical analysis, the data were analyzed by a one-way analysis of variance (ANOVA), followed by the Student-Newman-Keuls *post hoc* test for multiple comparisons. A significant difference was defined as a *p* value of <0.05.

## 4. Conclusions

Our studies on *Sinularia gaweli* for the extraction of additional natural substances, have led to the isolation of two eudesmane sesquiterpenoids, verticillatol (**1**) and 5α-acetoxy-4(14)-eudesmene-1β-ol (**2**) and two cembranoids, (–)-leptodiol acetate (**3**) and sinulacembranolide A (**4**) and compounds **2**–**4** are new isolates. Terpenoid **4** is potentially anti-inflammatory and may become a lead compound in marine anti-inflammatory drug development. The octocoral *Sinularia gaweli* will be transplanted to culturing tanks located in the National Museum of Marine Biology & Aquarium, Taiwan, for extraction of additional natural products to establish a stable supply of bioactive material.

## References

[1] Chen W.-T., Li Y., Guo Y.-W. (2012). Terpenoids of *Sinularia* soft corals: Chemistry and bioactivity. Acta Pharm. Sin. B.

[2] Rocha J., Peixe L., Gomes N.C.M., Calado R. (2011). Cnidarians as a source of new marine bioactive compounds—An overview of the last decade and future steps for bioprospecting. Mar. Drugs.

[3] Yen W.-H., Su Y.-D., Chang Y.-C., Chen Y.-H., Chen Y.-H., Dai C.-F., Wen Z.-H., Su J.-H., Sung P.-J. (2013). Sinulanorcembranolide A, a novel norcembranoidal diterpene from the octocoral *Sinularia gaweli*. Tetrahedron Lett..

[4] Hu L.-C., Yen W.-H., Su J.-H., Chiang M.Y.-N., Wen Z.-H., Chen W.-F., Lu T.-J., Chang Y.-W., Chen Y.-H., Wang W.-H. (2013). Cembrane derivatives from the soft corals, *Sinularia gaweli* and *Sinularia flexibilis*. Mar. Drugs.

[5] Yen W.-H., Chen W.-F., Cheng C.-H., Dai C.-F., Lu M.-C., Su J.-H., Su Y.-D., Chen Y.-H., Chang Y.-C., Chen Y.-H. (2013). A new 5α, 8α-epidioxysterol from the soft coral *Sinularia gaweli*. Molecules.

[6] Hong V.D., Tan G.T., Zhang H.-J., Tamez P.A., Hung N.V., Cuong N.M., Soejarto D.D., Fong H.H.S., Pezzuto J.M. (2002). Natural anti-HIV agents–part I: (+)-Demethoxyepiexcelsin and verticillatol from *Litsea verticillata*. Phytochemistry.

[7] Kitajima J., Suzuki N., Satoh M., Watanabe M. (2002). Sesquiterpenoids of *Torilis japonica* fruit. Phytochemistry.

[8] Díaz-Marrero A.R., Porras G., Cueto M., D’Croz L., Lorenzo M., San-Martín A., Darias J. (2009). Leptogorgolide, a biogenetically interesting 1,4-diketo-cembranoid that reinforces the oxidation profile of C-18 as taxonomical marker for octocorals. Tetrahedron.

[9] Li Y., Pattenden G. (2011). Perspectives on the structural and biosynthetic interrelationships between oxygenated furanocembranoids and their polycyclic congeners found in corals. Nat. Prod. Rep..

[10] Palframan M.J., Pattenden G. (2013). Searching for radical intermediates and pathways implied in the biosynthesis of some polycyclic cembranoids. A new plausible mechanism for the origin of sinulanorcembranolide A in the coral *Sinularia gyrosa*. Tetrahedron Lett..

[11] Rodríguez A.D., Li Y., Dhasmana H., Barnes C.L. (1993). New marine cembrane diterpenoids isolated from the Caribbean gorgonian *Eunicea mammosa*. J. Nat. Prod..

[12] Huang S.-Y., Chen N.-F., Chen W.-F., Hung H.-C., Lee H.-P., Lin Y.-Y., Wang H.-M., Sung P.-J., Sheu J.-H., Wen Z.-H. (2012). Sinularin from indigenous soft coral attenuates nociceptive responses and spinal neuroinflammation in carrageenan-induced inflammatory rat model. Mar. Drugs.

[13] Jean Y.-H., Chen W.-F., Sung C.-S., Duh C.-Y., Huang S.-Y., Lin C.-S., Tai M.-H., Tzeng S.-F., Wen Z.-H. (2009). Capnellene, a natural marine compound derived from soft coral, attenuates chronic constriction injury-induced neuropathic pain in rats. Br. J. Pharmacol..

[14] Jean Y.-H., Chen W.-F., Duh C.-Y., Huang S.-Y., Hsu C.-H., Lin C.-S., Sung C.-S., Chen I.-M., Wen Z.-H. (2008). Inducible nitric oxide synthase and cyclooxygenase-2 participate in anti-inflammatory and analgesic effects of the natural marine compound lemnalol from Formosan soft coral *Lemnalia cervicorni*. Eur. J. Pharmacol..

